# Liraglutide attenuates type 2 diabetes mellitus-associated non-alcoholic fatty liver disease by activating AMPK/ACC signaling and inhibiting ferroptosis

**DOI:** 10.1186/s10020-023-00721-7

**Published:** 2023-09-28

**Authors:** Tingli Guo, Wenhui Yan, Xin Cui, Na Liu, Xiaotong Wei, Yuzhuo Sun, KeXin Fan, Jieyun Liu, Yuanyuan Zhu, Zhuanzhuan Wang, Yilei Zhang, Lina Chen

**Affiliations:** 1 Department of Pharmacology, School of Basic Medical Sciences, Xi’an Jiaotong University Health Science Center, No. 76 Yanta West Road, Xi’an, 710061 Shaanxi People’s Republic of China; 2 Department of Biochemistry and Molecular Biology, School of Basic Medical Sciences, The Institute of Molecular and Translational Medicine, Xi’an Jiaotong University Health Science Center, No. 76 Yanta West Road, Xi’an, 710061 Shaanxi People’s Republic of China; 3 Institute of Cardiovascular Sciences, Translational Medicine Institute, Xi’an Jiaotong University Health Science Center, Xi’an, 710061 Shaanxi China; 4 Key Laboratory of Environment and Genes Related to Diseases, Xi’an Jiaotong University, Ministry of Education, Xi’an, 710061 Shaanxi China; 5 Department of Endocrinology, The First Affiliated Hospital of Xi’an Jiaotong University, Xi’an, 710061 Shaanxi China; 6 Department of Thoracic Surgery, The First Affiliated Hospital of Xi’an Jiaotong University, Xi’an, 710061 Shaanxi China

**Keywords:** Liraglutide, Type 2 diabetes mellitus, Non-alcoholic fatty liver disease, Lipid peroxidation, Ferroptosis

## Abstract

**Background:**

Non-alcoholic fatty liver disease (NAFLD) is one of the most common complications of type 2 diabetes mellitus (T2DM). The pathogenesis of NAFLD involves multiple biological changes, including insulin resistance, oxidative stress, inflammation, as well as genetic and environmental factors. Liraglutide has been used to control blood sugar. But the impact of liraglutide on T2DM-associated NAFLD remains unclear. In this study, we investigated the impact and potential molecular mechanisms of inhibiting ferroptosis for liraglutide improves T2DM-associated NAFLD.

**Methods:**

Mice were fed on high-fat-diet and injected with streptozotocin to mimic T2DM-associated NAFLD and gene expression in liver was analysed by RNA-seq. The fast blood glucose was measured during the period of liraglutide and ferrostatin-1 administration. Hematoxylin and eosin staining was used to evaluate the pathological changes in the liver. The occurrence of hepatic ferroptosis was measured by lipid peroxidation in vivo. The mechanism of liraglutide inhibition ferroptosis was investigated by in vitro cell culture.

**Results:**

Liraglutide not only improved glucose metabolism, but also ameliorated tissue damage in the livers. Transcriptomic analysis indicated that liraglutide regulates lipid metabolism related signaling including AMPK and ACC. Furthermore, ferroptosis inhibitor rather than other cell death inhibitors rescued liver cell viability in the presence of high glucose. Mechanistically, liraglutide-induced activation of AMPK phosphorylated ACC, while AMPK inhibitor compound C blocked the liraglutide-mediated suppression of ferroptosis. Moreover, ferroptosis inhibitor restored liver function in T2DM mice in vivo.

**Conclusions:**

These findings indicate that liraglutide ameliorates the T2DM-associated NAFLD, which possibly through the activation of AMPK/ACC pathway and inhibition of ferroptosis.

**Supplementary Information:**

The online version contains supplementary material available at 10.1186/s10020-023-00721-7.

## Background

Type 2 diabetes mellitus (T2DM) is the most common type of diabetes, accounting for around 90% of all diabetes cases. Consistently high blood glucose levels can lead to serious diseases affecting the heart (cardiovascular disease), kidney (diabetic nephropathy), liver (non-alcoholic fatty liver disease, NAFLD), nerve (diabetic neuropathy) and eyes (diabetic retinopathy), et al. (Alkethiri et al. 2021; Damanik and Yunir 2021; DeFronzo et al. 2015; Tilg et al. 2017). A meta-analysis showed that the overall prevalence of NAFLD among patients with type 2 diabetes mellitus is 55.5%(Younossi et al. 2019). The coexist between T2DM and NAFLD act synergistically to increase the risk of adverse clinical outcomes. NAFLD is a heterogeneous metabolic disease, and the “multiple-hit” theory for the development of NAFLD is widely accepted, including lipid accumulates in the liver, insulin resistance, oxidative stress, inflammation, genetic and environmental factors, et al. (Berlanga et al. 2014; Juanola et al. 2021; Perla et al. 2017; Salvoza et al. 2022). Dysfunction of energy homeostasis is thought to be an important factor in driving changes in a wide range of metabolic diseases (Carling 2017). The AMP-activated protein kinase (AMPK), a central mediator of the cellular response to energetic stress, is a highly conserved master regulator of metabolism (Herzig and Shaw 2018). AMPK activity is decreased during obesity and hyperglycemia, and its activation has positive effects in many diseases such as insulin resistance, diabetes, obesity, cancer and Alzheimer's (Canbolat and Cakıroglu 2022). AMPK is a classical phosphorylation upstream kinase of Acetyl-CoA carboxylase (ACC). ACC are enzymes that catalyze the carboxylation of acetyl-CoA to produce malonyl-CoA, and plays a crucial role in fatty acid metabolism (Brownsey et al. 1997). ACC enzyme have two isoforms: ACC1 is primarily expressed in lipogenic tissues, especially in liver and adipose; Higher levels of ACC2 express in lipogenic and oxidative tissues, including heart, skeletal and muscle (Harada et al. 2007). AMPK phosphorylates ACC1/2 on serine residues (Ser79/212), leading to suppression of ACC activity (Pang et al. 2021). Liver-specific AMPK knockout aggravated liver damage in mouse NASH models (Zhao et al. 2020). AMPK activation may be an effective strategy in the exploration of NAFLD treatment.

Ferroptosis, a recently identified non-apoptotic form of regulated cell death that is characterized by iron-dependent lipid peroxidation, is distinct from apoptosis, necrosis, and autophagy in morphology, biochemistry and genetics (Dixon et al. 2012; Hirschhorn and Stockwell 2019). In recent years, accumulating evidence indicates that ferroptosis is involved in the progression of NAFLD (Gautheron et al. 2020; Wang et al. 2022b). Lipid peroxidation is one of the hallmarks of ferroptosis. Malondialdehyde and 4-hydroxynonenal (4-HNE) levels are increased in patients with NAFLD (Loguercio et al. 2001). On the contrary, vitamin E improves liver injury in patients with NAFLD via decreasing lipid peroxidation (Podszun et al. 2020). Ferroptosis was confirmed to be the initial cell death process that triggers nonalcoholic steatohepatitis (NASH) (Tsurusaki et al. 2019). Liproxstatin-1, a ferroptosis inhibitor, repressed hepatic lipid peroxidation and its associated cell death, resulting in decreased NASH severity (Qi et al. 2020). However, how ferroptosis regulates NAFLD remains largely unknown.

Liraglutide is a glucagon-like peptide-1(GLP-1) analog, which is a 30-amino acid peptide hormone secreted by the intestinal epithelial endocrine L-cells (Holst 2007). GLP-1 binding to GLP-1 receptor (GLP-1R) can exert a variety of physiological effects, including suppression of food intake and postprandial glucagon secretion, delayed gastric emptying, lowering fast blood glucose (FBG) and body weight (BW) (Jorsal et al. 2016). These actions have been clinically approved for treatment of T2DM and obesity (Juhl et al. 2002). In addition, liraglutide is beneficial for cardiovascular (Mikhail 2019) and shows excellent neuroprotective capacities by activating AMPK pathway (Song et al. 2022b). Liraglutide binding to GLP-1 receptor in extrahepatic organs improve hepatic lipid homeostasis via stimulating hepatic fibroblast growth factor 21 (FGF21) (Liu et al. 2021). A recent study has reported that liraglutide alleviates diabetic-associated cognitive deficit via reducing oxidative stress, lipid peroxidation and iron overload and further inhibiting ferroptosis (An et al. 2022). AMPK has been shown to control lipid metabolism and negatively regulate ferroptosis (Lee et al. 2020; Li et al. 2020a; Nguyen et al. 2016). Here, we found that ferroptosis plays a key role in T2DM-associated NAFLD, and the protective effects of liraglutide against the progression of NAFLD is related to underlying inhibitory ferroptosis mechanism of AMPK activation.

## Materials and methods

### Animal study

The Ethics Committee on the Use and Care of Animals at Xi’an Jiaotong University approved the study protocol. The animals (male C57BL/6J mice, aged 7–8 weeks, weighing 18–22 g) received humane care according to the criteria outlined in the Guide for the Care and Use of Laboratory Animals prepared by the National Academy of Sciences and published by the National Institutes of Health. The mice were randomized into 2 groups, fed by a high-fat diet (HFD) or normal diet for 8 weeks. HFD (60% fat, 20% protein and 20% carbohydrates) purchased from Jiangsu Synergy Co., Ltd. Streptozotocin (STZ, 60 mg/kg body weight; Sigma-Aldrich, USA) was then intraperitoneally injected to HFD-fed mice (for 3 consecutive days, 60 mg/kg body weight) to further induce T2DM model. After following fasting for 12 h, animals with fasting blood glucose (FBG) levels ranging from 15 to 25 mmol/L measured by Ruidian Performa glucometer (Shenzheng Ruidian Technology Co., Ltd, Shenzheng, China). 3 days after STZ injection were considered diabetic. Subsequently, all T2DM mice and the normal mice were randomly divided into 3 subgroups as follows: (1) normal diet group (control group); (2) HFD diet model (T2DM model group) (3) liraglutide (Lira, Novo Alle, DK-2880 Bagsvaerd, Denmark) intervention group or ferrostatin-1 (Fer-1, MedChemExpress, Shanghai, China) intervention group. Lira (300 mg/kg/day) was administrated three times a week for 6 weeks. Fer-1 group was intraperitoneal injected with ferrostatin-1 at 1 mg/kg daily for 6 weeks. Meanwhile, the mice in the control group were received injection of equivalent volume of normal saline for 6 weeks. All animals were maintained under standardized conditions (consistent temperature 22 ± 1 °C, relative humidity 50 ± 10%, and 12 h light/dark cycle). Animals were provided with a corresponding diet and allowed free access to tap water. Body weight (BW) and FBG were measured weekly.

### Glucose tolerance test (IPGTT and insulin tolerance test (ITT)

Mice were injected intraperitoneally with glucose (2 g/kg body weight) when the mice were fasted for 12 h at the 5th week during the drug administration. Blood samples were taken by tail prick at 0, 30, 60, and 120 min for the measurement of blood glucose levels using glucometer. Area under the curve (AUC) of IPTGG was calculated. ITT was carried out in the next day. The mice which were fasted 6 h were injected with insulin (0.75 U/kg body weight) (Jiangsu Wanbang Biochemistry Medicine Co. Ltd., Xuzhou, China). Glucose was measured at 0, 15, 30, 60 and 120 min after insulin injection. Area under the curve (AUC) of ITT was calculated.

### Serum analyses

The serum level of total cholesterol (TC), triglyceride (TG), low density lipoprotein-cholesterol (LDL-C), alanine aminotransferase (ALT) and aspartate aminotransferase (AST) were measured according to instructions of reagent kits (Nanjing jiancheng Bioengineering Institute, Nanjing, China).

### Histopathologic examination

Liver tissues were fixed with 4% paraformaldehyde and then embedded in paraffin. Embedded tissue was cut to 4-μm thickness which were then stained with hematoxylin–eosin (H&E) kit (Servicebio, Wuhan, China) for histological assessment. Oil-red O staining was conducted with frozen liver tissues using a commercially available Oil-Red O stain kit (Servicebio, Wuhan, China) according to the instruction. For immunohistochemistry (IHC), the deparaffinized sections were subjected to heat-mediated antigen retrieval and blocked in 3% H_2_O_2_ for 20 min. The sections were stained overnight with a primary anti-cleaved caspase 3 antibody (1:200; CST, #9664), anti-4HNE antibody (1:200, Abcam, ab46545) after blocking with bovine serum albumin for 1 h at room temperature. The sections were incubated with a secondary antibody (1:5000; HRP, Donkey Anti-Goat IgG, Proteintech, Wuhan, China) after washing with PBS for 15 min at room temperature. Then, the colour were developed with a 3,3′-diaminobenzidine tetrahydrochloride (DAB; Servicebio, Wuhan, China) after PBS wash, and then the nuclei were restrained with Harris hematoxylin for 3 min. After dehydrating and sealing, images were obtained using a microscope (BX51, Olympus, Tokyo, Japan).

### RNA-sequencing

RNA-seq was performed on triplicate sample from liver of three group mice (Control, Model and Lira). The total RNA was extracted using Trizol (Ambion, USA). RNA quality was verified using Agilent 2100 Bio-analyzer (Agilent Technologies, Santa Clara, CA). RNA sequencing libraries were generated using Agilent High Sensitivity DNA Kit, and sequenced on an Illumina Novaseq 6000 platform. Sequencing was performed at Bioprofile Co. Ltd (Shanghai, China). The filtered reads were mapped to the mouse genome reference sequence (GRCm39.dna.toplevel.fa Ensembl release103) using HISAT2. Gene expression levels were calculated by the fragments per kilobase of transcript per million mapped reads (FPKM) values. Genes were considered differentially expressed when ∣log2 (fold change)∣ > 1.5 and P-value < 0.05.

### Real-time PCR

Total RNA was extracted from liver of mouse using TRI Gen (GenStar, China), and reverse-transcribed with the ABscript II cDNA First Strand Synthesis Kit (ABclonal, China). Real-time PCR was conducted by using ABScript III RT Master Mix for qPCR (ABclonal, China). β-actin was used as an internal control and Relative expression of target mRNA was calculated based on the 2^−ΔΔ^Ct comparative method. The primer sequences used are listed in Table 1.

**Table 1:** Sequences of primers for real-time PCR

Gene	Forward primer	Reverse primer
ACACA	ATGGGCGGAATGGTCTCTTTC	TGGGGAACCTTGTCTTCATCAT
HMGCR	TAGCACTGGTCCAGGAAACC	CACAGGAACAAGGCACACAG
LDLR	ACCTGCCGACCTGATGAATCC	GCAGTCATGTTCACGGTCACA
β-actin	TGTGACGTTGACATCCGTAA	GCTAGGAGCCAGAGCAGTAA

### Cell culture studies

The human liver cancer cell lines HepG2, Hep3B and PLC cell lines obtained from the Cancer Research Institute, Xi’an Jiaotong University. HepG2, Hep3B and PLC cells were cultured in Dulbecco’s modified Eagle medium (Gibco, US) in a 37 ^◦^C incubator with an atmosphere of 5% CO_2_. All the medium supplemented with 10% fetal bovine serum (Excell Bio, China) and 1% penicillin–streptomycin (Solarbio, Beijing, China).

### Cell viability assay

Cell viability was measured as described previously using the Cell Counting Kit-8 (CCK8) (Enogene, Nanjing, China). In brief, cells were seeded in 96-well plates at a density 4 × 10^3^–8 × 10^3^ cells per well and incubated for 24 h. Then, the cells were exposed to different treatment. Cells were treated with glucose (Solarbio, Beijing, China)., ferroptosis inducers, erastin (Selleckchem, Houston, USA), or RSL3 (Selleckchem, Houston, USA); Liraglutide; cell death inhibitors, including ferrostatin-1 (Selleckchem, Houston, USA), necrostatin-1 s (Selleckchem, Houston, USA), or Z-VAD-fmk (Selleckchem, Houston, USA); AMPK inhibitor, compound C (MedChemExpress, USA); After that, the medium in each well was replaced with 100 µl fresh medium containing 10 µl CCK8 reagent. After incubation for 1 h at 37 °C, 5% CO_2_ incubator. The absorbance at a wavelength of 450 nm was measured using a Multiskan Spectrum (Thermo Scientific, Waltham, MA, USA).

### Measurement of intracellular ferrous iron level (Fe^2+^)

The intracellular Fe^2+^ level was determined using RhoNox-1 (HY-D1533, MCE). First, cells were cultured on sterile coverslips. Then, remove the coverslip from the medium and aspirate excess medium. Furthermore, add 100uL of working solution, gently shake it to completely covers the cells, and then incutate at 37℃ for 1 h. At last, wash twice with medium, 5 min each time and observe by fluorescence microscopy.

### Western blotting

Western blotting to analyze protein expression was performed as previously described (Yi et al. 2022). Briefly, liver tissues and cell pellets were lysed using RIPA lysis buffer (Millipore) and the protein concentration was determined by a Pierce BCA kit (Thermo, Waltham, MA, USA). The primary antibodies and concentrations used for western blotting were: phospho-AMPKα (Thr172, 1:1000, 2535, Cell Signaling, Boston, USA), AMPKα (1:1000, 5832, Cell Signaling, Boston, USA), phospho-ACC (S79, 3661, 1:1000, Cell Signaling, Boston, USA), ACC (1:1000, 3662, Cell Signaling, Boston, USA), phospho-S6 (Ser240/244, 1:1000, 2215, Cell Signaling, Boston, USA), S6 (1:1000, 2217, Cell Signaling, Boston, USA), phospho-S6K (Thr229/389, 1:1000, 9202, Cell Signaling, Boston, USA), S6K (1:1000, 9205, Cell Signaling, Boston, USA), TFRC (1:1000, A5865, Abclonal, Wuhan, China), ACSL4 (sc-271800, 1:1000, Santa Cruz), SLC7A11 (12691S, 1:1000, Cell Signaling, Boston, USA), GPX4 (52455S, 1:1000, Cell Signaling, Boston, USA), beta actin (TA811000S, 1:2000, Origene), Tubulin (80762-1-RR, 1:10,000, Proteintech, Wuhan, China). The secondary antibodies used were: horseradish peroxidase-conjugated anti-rabbit IgG (Cell Signaling Technology, 1:5000 dilution, Wuhan, China), horseradish peroxidase-conjugated anti-mouse IgG (Cell Signaling Technology, 1:5000 dilution). Proteins were visualized with the ECL Western blotting substrate (32,109, ThermoScientific, USA).

### Statistical analysis

Data are presented as means ± standard deviation (SD), with at least 3 independent biological replicates in each group. The difference between the two groups was tested by using unpaired Student’s t test with GraphPad Prism 8 (GraphPad Software, Inc.). One-way analysis of variance or two-way analysis of variance was used to determine the significance among three or more groups replicates. (*P < 0.05, **P < 0.01, ***P < 0.001, ****P < *0.0001*, *ns*, non-significant). The band intensity of western blotting and fluorescence intensity of Fe^2+^ are measured by using Image J. All samples from cells were collected from at least 3 independent biological replicates.

## Results

### Liraglutide improves glucose tolerance and insulin tolerance in T2DM mice

**Fig. 1:**
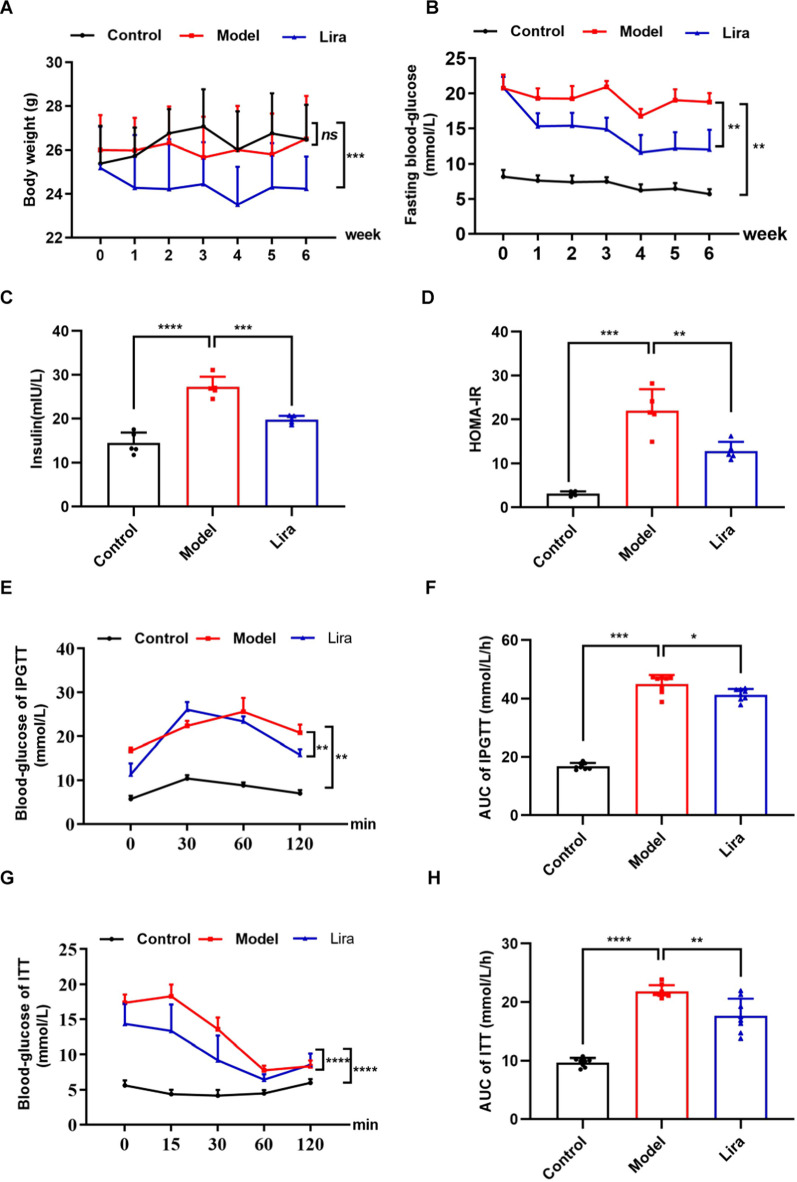
Liraglutide (Lira) maintains glucose homeostasis of T2DM mice. The effect of liraglutide on **A** body weight (BW) (n = 8), **B** fast blood glucose (FBG) (n = 8), **C** insulin (n = 5), **D** HOMA-IR (n = 5), **E** IPGTT (n = 8), **F** AUC of IPGTT(n = 8), **G** ITT (n = 8) and (**H**) AUC of ITT (n = 8) of T2DM mice. Data are expressed as mean ± SD. ^*^P < 0.05, ^**^P < 0.01, ^***^P < 0.001, ^****^P < 0.0001, ns means no significant difference

To investigate the role of liraglutide on the regulation of metabolic disorders, T2DM model mice were administrated with liraglutide for 6 weeks. As shown in Fig. 1A, liraglutide treatment for six weeks had a significant decrease on the BW of the T2DM mice. Liraglutide treatment also significantly reduced fasting blood glucose (FBG) of T2DM mouse model (Fig. 1B). The model mice showed a significant increase in insulin levels (Fig. 1C) and HOMA-IR (Fig. 1D) of serum, while liraglutide treatment leaded to a significant reduction of insulin and HOMA-IR. Furthermore, T2DM mice seemed to have an impaired tolerance to glucose (Fig. 1E) and an impaired tolerance to insulin (Fig. 1G), while liraglutide-treated mice significantly improved glucose tolerance and insulin tolerance. The area under the curve (AUC) of IPGTT (Fig. 1F) and AUC of ITT (Fig. 1H) also showed the similar results. Above results suggest that liraglutide improves glucose metabolism and alleviates insulin resistance of T2DM mice.

### Liraglutide alleviates liver damage and hepatic lipid accumulation of T2DM mice

**Fig. 2:**
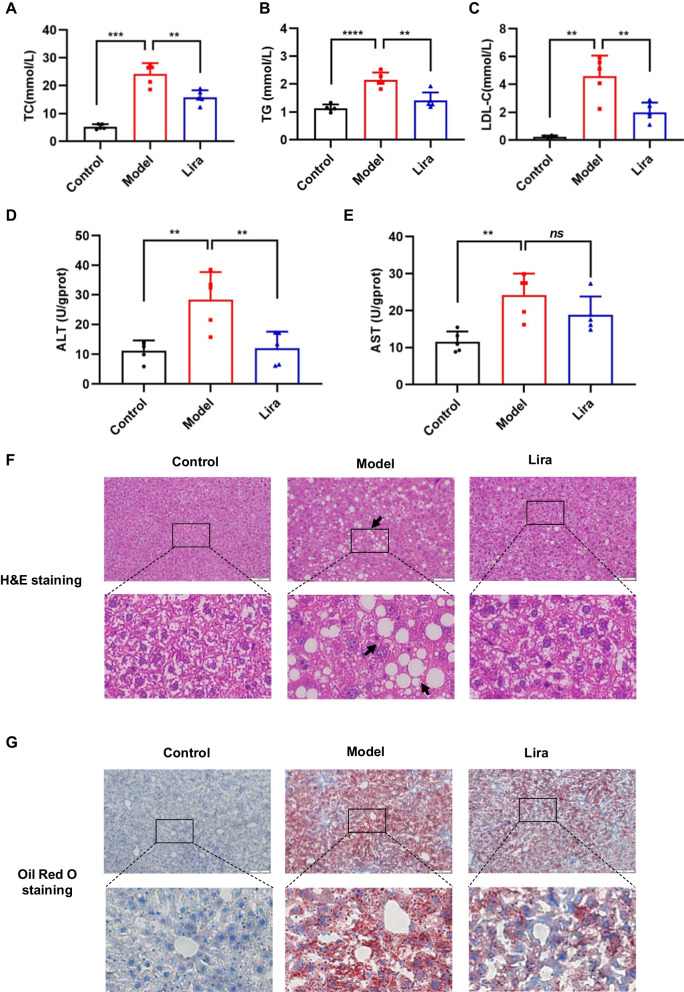
The effects of liraglutide on liver histology, liver injury and lipid accumulation of T2DM mice. The serum levels of TC (**A**), TG (**B**) and LDL-C (**C)** in different groups. The serum levels of ALT (**D**) and AST (**E**) were measured. **F** Representative images of H&E staining of liver (scale bar, 100 μm). **G** Representative images of Oil Red O staining of liver (scale bar, 100 μm). Data are expressed as mean ± SD. n = 5. ^*^P < 0.05, ^**^P < 0.01, ^***^P < 0.001, ^****^P < 0.0001, ns means no significant difference

It is generally known that disorder of lipid metabolism is one of the most important features of NAFLD. Here, we examined the effect of liraglutide on blood lipids, such as total cholesterol (TC), triglyceride (TG), low density lipoprotein-cholesterol (LDL-C) of serum. It’s shown that T2DM mice have a significant increase in TC (Fig. 2A), TG (Fig. 2B) and LDL-C (Fig. 2C) contents of serum. Liraglutide treatment remarkably decreased levels of TC, TG and LDL-C compared to the T2DM mouse model (Fig. 2A–C). To exam whether liraglutide treatment could improve liver damage in T2DM mice, alanine aminotransferase (ALT) and aspartate aminotransferase (AST) were measured. The results showed that liraglutide significantly reduced ALT level (Fig. 2D) of T2DM mice, but not AST level (Fig. 2E). Histopathological examination by H&E staining of the liver tissue showed ballooning of hepatocytes of T2DM mice was significantly alleviated after liraglutide administration (Fig. 2F). To quantify the lipid accumulation of liver, we performed Oil Red O staining. Lipid droplet accumulation in the livers of T2DM mice were significantly enhanced compared to control mice. After liraglutide treatment, oil red O-positive areas were significantly reduced (Fig. 2G). These findings prompt us to further study the underlying functional mechanisms of liraglutide.

### Liraglutide regulates the expression of genes involved in the lipid metabolism

**Fig. 3:**
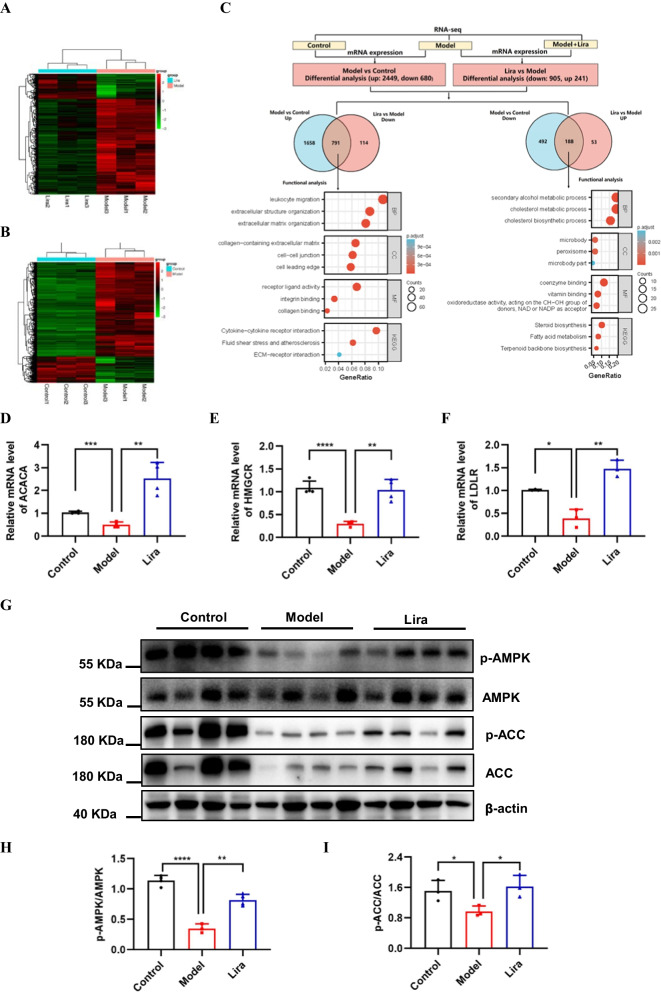
Liraglutide regulates lipid metabolism. **A** Heatmap shows differential expressed genes in hepatic tissue between control and model mice. Low expression is depicted in green, and high expression is depicted in red (n = 3). **B** Heatmap shows differential expressed genes in hepatic tissue between model and liraglutide mice. Low expression is depicted in green, and high expression is depicted in red (n = 3). **C** Gene ontology (GO) and Kyoto Encyclopedia of Genes and Genomes (KEGG) enrichment analyses the overlap genes, which are overlapped between upregulated 2449 genes (Model vs Control) and downregulated 905 genes (Lira vs Model), between downregulated 680 genes (Model vs Control) and upregulated 241 genes (Lira vs Model). **D**–**F** ACACA (n = 4), HMGCR (n = 4) and LDLR (n = 3) mRNA levels in three group mice were analyzed by RT-PCR. **G** Phosphorylated AMPK levels and phosphorylated ACC protein levels in the three group mice were analyzed by Western blotting (n = 4). **H–I** Quantification of G. Data are expressed as mean ± SD. ^*^P < 0.05, ^**^P < 0.01, ^***^P < 0.001, ^****^P < 0.0001

To explore the mechanisms underlying of liraglutide protection against NAFLD, we performed RNA-seq analysis of liver samples. The mRNA expression levels of all genes are listed in Additional file 1: Table S1. As shown in Fig. 3A–C 2449 genes were up-regulated and 680 genes were down-regulated in the T2DM model group compared with the normal control group. Besides, 241 genes were up-regulated and 905 genes were down-regulated in the liraglutide-treatment group compared with the model group in Fig. 3B, C (fold change > 1.5, FDR < 0.05). To identify the biological processes associated with the differential expression genes, we used Gene Ontology (GO) and Kyoto Encyclopedia of Genes and Genomes (KEGG) pathway analyses. Notably, integration of both up-regulated 2449 genes of Model vs Control and down-regulated 905 genes of Lira vs Model identified 791 overlap genes that are related with collagen-containing extracellular matrix, collagen-containing extracellular matrix and collagen binding, et al. (Fig. 3B). Integration of both down-regulated 680 genes of Model vs Control and up-regulated 241 genes of Lira vs Model identified 188 overlap genes that are generally associated with cholesterol metabolic process, cholesterol biosynthetic process, steroid biosynthetic process, steroid metabolic process and fatty acid metabolism et al. (Fig. 3B). Further analysis by RT-PCR revealed that numerous genes involved in cholesterol metabolic process, including acetyl-CoA carboxylase alpha (ACACA, also known as ACC), 3-hydroxy-3-methylglutaryl-CoA reductase (HMGCR) and low density lipoprotein receptor (LDLR), which were drastically upregulated in liraglutide-treatment group (Fig. 3D, F). The AMP-activated protein kinase (AMPK) is a key regulator of nutrient metabolism, and ACC is a bona fide substrate of AMPK that controls lipid metabolism. Thus, we further examined the protein and activation levels of AMPK and ACC. Western blotting results revealed that T2DM model decreased the phosphorylation levels of AMPK and ACC, but liraglutide-treatment significantly restored p-AMPK and p-ACC in the liver of T2DM mice (Fig. 3G–I). These results suggest that liraglutide possibly regulates lipid metabolism during NAFLD progression in mice through AMPK/ACC axis.

### Liraglutide alleviates HG-induced sensitivity to ferroptotic cell death

**Fig. 4:**
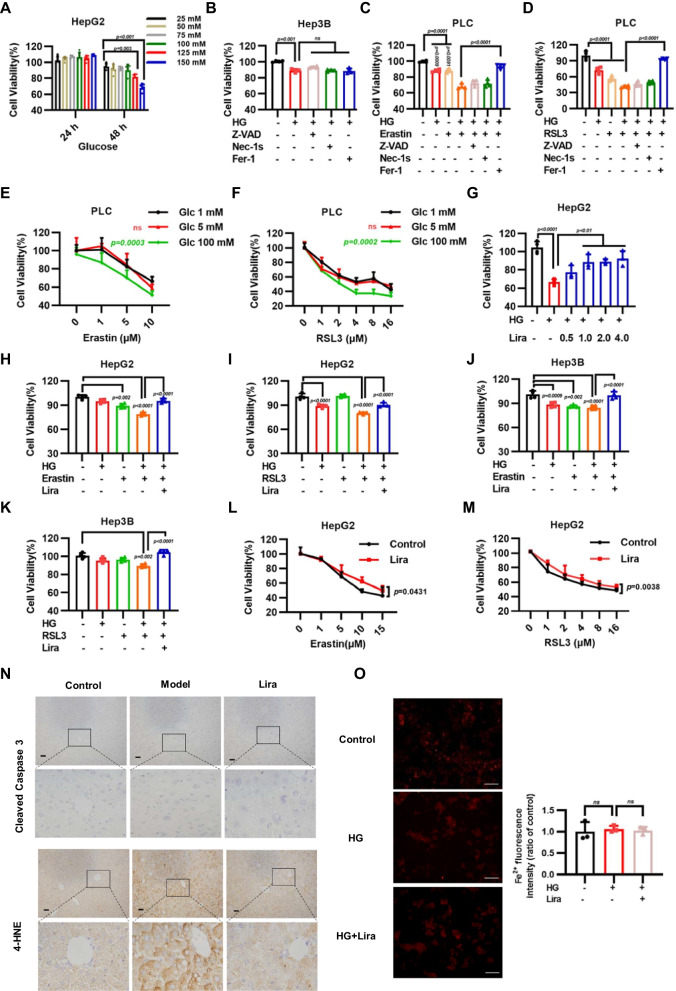
Liraglutide inhibits sensitivity of high glucose on ferroptosis. **A** Cell viability in HepG2 cells treated with indicated concentrations of high glucose (HG) for 24 h and 48 h. **B** Cell viability in Hep3B cells treated with HG (125 mM, 48 h) and combined with 5 µM Z-VAD-FMK (Z-VAD, 24 h), 2 µM Necrostatin-1 s (Nec-1 s, 24 h) and 5 µM ferrostatin-1 (Fer-1, 24 h)**.** Bar graphs showing cell viability in indicated cells treated with HG (125 mM, 48 h) or erastin (10 µM, 24 h) **(C)**, RSL3 (1.0 µM, 24 h) **D** and combined with 5 µM Z-VAD-FMK (Z-VAD, 24 h), 2 µM Necrostatin-1 s (Nec-1 s, 24 h) and 5 µM ferrostatin-1 (Fer-1, 24 h). PLC cells viability were measured with 1 mM, 5 mM, 100 mM glucose (48 h) combined with erastin (24 h) **E** or RSL3 (24 h) **(F)**. **G** Cell viability measured in HepG2 cells treated with 125 mM HG and different concentrations of liraglutide for 48 h. **H**–**I** HepG2 and Hep3B cells were treated with 100 mM HG, erastin (5 μM) and combined with 1.0 μM liraglutide for 24 h. **J**, **K** HepG2 and Hep3B cells were treated with 100 mM HG, RSL3 (0.5 μM) and combined with 1.0 μM liragluide for 24 h. Cell viability was determined by CCK-8 assays. **L** Cell viability measured in HepG2 cells treated with different concentrations of erastin (24 h) combined with 2.0 μM liraglutide (48 h). **M** Cell viability measured in HepG2 cells treated with different concentrations of RSL3 (24 h) combined with 2.0 μM liraglutide (48 h). **N** Representative images from immunohistochemical staining of liver tissue microarray for Cleaved caspase-3 and 4-HNE (scale bar, 50 μm). **O** Representative Fe^2+^ fluorescence images of HepG2 cell (scale bar, 100 μm) Data are expressed as mean ± SD, n = 4

High glucose level is a detrimental factor that promotes pathogenesis in T2DM mice. To determine the impact of HG on liver cells, HepG2 cells were exposed to different concentrations of glucose. The results showed that high concentration of glucose like 100 mM or 125 mM glucose significantly inhibited cell growth after treatment for 48 h (Fig. 4A). Lipid metabolism is critical for ferroptotic cell death, which plays an important role in variety of diseases. To verify whether HG regulates ferroptosis, we firstly found that ferroptosis inhibitor Fer-1 couldn’t rescue HG-induced decreased cell viability (Fig. 4B). Whether the effects of erastin-induced or RSL3-induced ferroptosis was enhanced by the addition of HG remain unclear. Notably, HG treatment combined with erastin or RSL3 indeed significantly reduced cell viabilities, which was largely reversed by the application of the ferroptosis inhibitor Fer-1, but not apoptosis inhibitor Z-VAD-FMK or necroptosis inhibitor Nec-1 s (Fig. 4C, D). Further experiments demonstrated that 100 mM glucose but not 5 mM or 1 mM glucose treatment significantly promoted cell death of PLC cells upon treated with different concentration of RSL3, suggesting that HG sensitizes cells to ferroptotic cell death (Fig. 4E, F). Next, we wonder whether Lira could abolish the cytotoxic effect caused by HG. Importantly, we found that liraglutide can promote cell survival in dose-dependent manner when HepG2 cells were exposed to 125 mM HG (Fig. 4G). In addition, liraglutide also significantly abolished the ferroptosis caused by combination treatment with HG and earstin or RSL3 in HepG2 and Hep3B cells (Fig. 4H, K). We also observed that liraglutide inhibits ferroptosis induced by erastin or RSL3 alone (Fig. 4L, M). Ferroptosis is characterized by overproduction of lipid peroxidation. Next, we conducted cleaved caspase 3 and 4-HNE immunohistochemistry (IHC) analysis to characterize apoptotic factors levels and lipid peroxidation levels in liver tissue of mouse. The results revealed increased 4-HNE staining, but not cleaved caspase 3 levels in liver tissue of T2DM mice. Conversely, liraglutide reduced T2DM-induced 4-HNE staining in liver (Fig. 4N). Intracellular ferrous iron level (Fe^2+^) was measured by RhoNox-1, a specific fluorescent probe for the detection Fe^2+^ levels of living cells, founding that there were no significant changes in ferrous ion levels after HG or liraglutide treatment (Fig. 4O). Taken together, our data support that HG enhanced sensitivity to ferroptotic cell death, while liraglutide could block ferroptosis induced by HG and ferroptosis-inducing agents like RSL3 and erastin.

### Liraglutide inhibits ferroptosis partly through AMPK-mediated phosphorylation of ACC

**Fig. 5:**
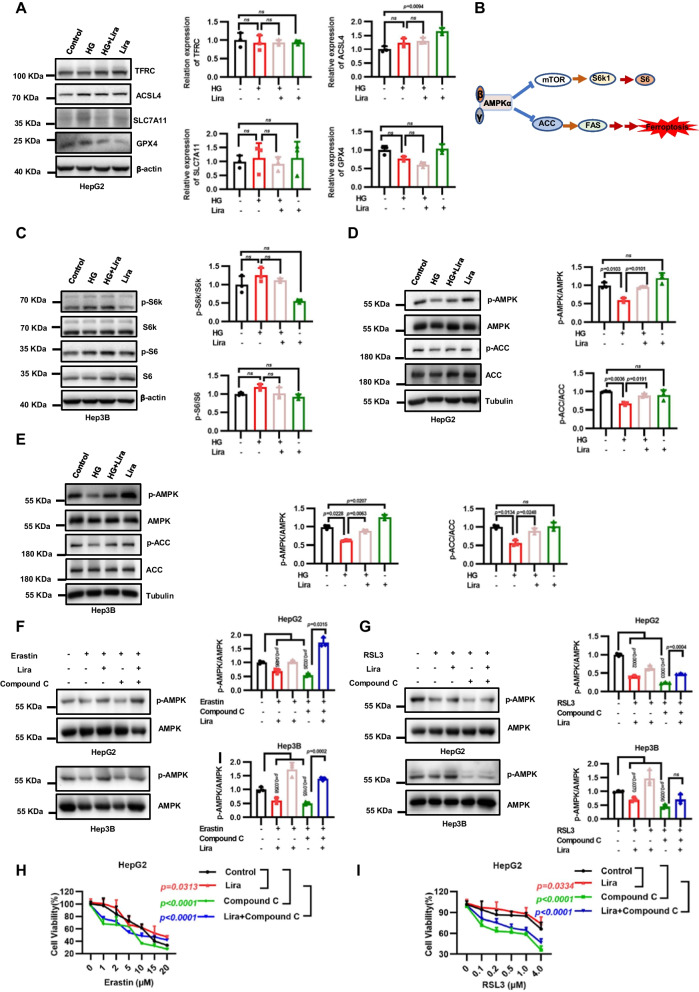
Liraglutide inhibits ferroptosis by activating AMPK/ACC signaling. **A** Western blotting show TFRC, ACSL4, SLC7A11 and GPX4 levels in HepG2 cells after 48-h incubation with 125 mM HG and 2.0 μM liraglutide. **B** Simplified schematic representation of the downstream pathway of AMPK.** C** Western blotting show phosphorylated S6K and phosphorylated S6 levels in HepG2 cells after 48-h incubation with 125 mM HG and 2.0 μM liraglutide. **D** Western blotting show phosphorylated AMPK levels and phosphorylated ACC levels in HepG2 cells after a 4-h incubation in glucose-free medium and a 4-h incubation with 125 mM HG, 2.0 μM liraglutide and combination. **E** Western blotting show phosphorylated AMPK levels and phosphorylated ACC levels in Hep3B cells after a 4-h incubation in glucose-free medium and a 4-h incubation with 125 mM HG, 2.0 μM liraglutide and combination. **F** Western blotting showing the levels of ACC (S79) and AMPK (T172) phosphorylation in HepG2 and Hep3B cells treated with 2.0 μM erastin for 12 h, or 5.0 μM Compound C for 12 h, 2.0 μM liraglutide for 48 h and the combination. **G** Western blotting showing the levels of ACC (S79) and AMPK (T172) phosphorylation in HepG2 and Hep3B cells treated with 1.0 μM RSL3 for 12 h, or 5.0 μM Compound C for 12 h, 2 μM liraglutide for 48 h and the combination. **H** Cell viability in HepG2 were measured treated as F. **I** Cell viability in HepG2 were measured treated as **G**. Data are expressed as mean ± SD, n = 4

Our aforementioned data revealed that liraglutide could inhibit ferroptosis in vitro, which leads us to further study the underlying mechanisms. First, we found that HG or liraglutide had little effect on key regulatory proteins for ferroptosis in HepG2 cells, including TFRC, ACSL4, SLC7A11 and GPX4 (Fig. 5A). In view of that liraglutide upregulated AMPK phosphorylation levels of liver in T2DM mice, we further verify the important role of AMPK in vitro. AMPK can inhibit the mTOR/S6K signaling pathway or promote the phosphorylation of ACC, both of which have been shown to regulate ferroptosis sensitivity (Fig. 5B). Our results showed that HG didn’t decrease the protein expression of p-S6K and p-S6, and liraglutide had no obvious effect on S6K and S6 (Fig. 5C). However, HG significantly decreased the level of p-AMPK and p-ACC. Moreover, liraglutide treatment could rescue the level of p-AMPK and p-ACC (Fig. 5D–E). To investigate whether liraglutide inhibits ferroptosis via activating AMPK, we showed that inactivating AMPK by compound C dramatically decreased p-AMPK level in liver cancer cell lines, and sensitized cancer cells to ferroptosis induced by erastin or RSL3, regardless of liraglutide treatment (Fig 5F–I). Together, these findings show that AMPK activation is essential for liraglutide to protect cells against ferroptosis.

### Ferroptosis inhibitor Fer-1 improves liver injury of T2DM mice

**Fig. 6:**
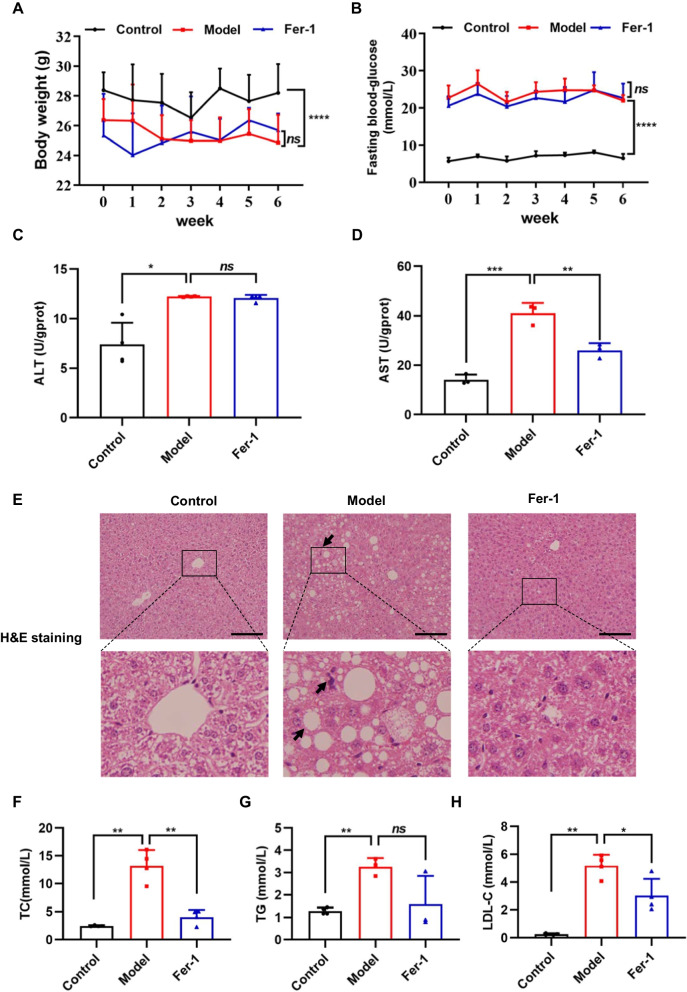
Ferrostatin-1 (Fer-1) prevents T2DM-induced liver injury. The effect of Fer-1 on **A** body weight (BW) (n = 6) and **B** fast blood glucose (FBG) (n = 6). **C**, **D** Serum levels of ALT and AST in mice were detected (n = 4). **E** Histopathological changes were examined by H&E staining. Scale bar = 100 μm. **F–H** Serum levels of TC, TG and LDL-C in mice were detected (n = 4). Data are expressed as mean ± SD, *P < 0.05, **P < 0.01, ***P < 0.001, ****P < 0.0001, ns means no significant difference

To further identify the involvement of ferroptosis in NAFLD, the therapeutic action of Fer-1 was further evaluated in vivo using T2DM-induced NAFLD mouse model. We found that Fer-1 treatment didn’t obvious affect BW (Fig. 6A) and FBG (Fig. 6B) of T2DM mice. The levels of AST and ALT of serum were measured, and the results indicated that the AST levels (Fig. 6D) was significantly decreased after Fer-1 treatment compared with T2DM mice, but not ALT levels (Fig. 6C). HE staining showed that the liver tissues of T2DM mice exhibited the greatest degree of injury compared with control mice, while Fer-1 administration markedly alleviated hepatic steatosis (Fig. 6E). Besides, Fer-1 markedly decreased.

TC, TG and LDL-C contents of serum in T2DM mice (Fig. 6F–H). These data indicate that ferroptosis might participate in the progression of NAFLD, while ferroptosis inhibitors might be therapeutically applied to treat NAFLD.

## Discussion

T2DM seems to accelerate the progression of liver disease in NAFLD (Bril and Cusi 2016). Liraglutide, an agonist of GLP-1R, was approved for treatment of T2DM in 2009 and chronic weight management in 2015 clinically (Iepsen et al. 2015). Previous study showed that liraglutide ameliorates glycometabolism and insulin resistance in diabetic mice (Chen et al. 2013). These findings were consistent with our results, which liraglutide reduces BW, decreases FBG levels, increases insulin secretion and improves impaired insulin tolerance and glucose tolerance of T2DM mice. In recent years, with the rapid research progress of GLP-1, liraglutide has gradually become a hot spot in the research field of NAFLD. Excessive hepatic lipid composition is an important factor for the progression of NAFLD. The present findings show that liraglutide improved liver injury and reduced lipid deposition of liver. These data indicated that liraglutide improve insulin resistance and attenuate hepatic lipid accumulation. Gut microbiota dysregulation plays a key role in the pathogenesis of T2DM and NAFLD(Ge et al. 2023), previous studies showed liraglutide can lead to gut microbiota modulations, such as a reduction of Proteobacteria and an increase of Akkermansia muciniphila in the HFD mouse(Moreira et al. 2018). However, the potential mechanism about that liraglutide improves NAFLD linked with gut microbiota deserves further investigation.

Further studies need to explore the mechanism of liraglutide protects against NAFLD. Our RNA-seq data showed that liraglutide reversed some lipid metabolism-related genes altered in NAFLD. The low-density lipoprotein receptor (LDLR), a key lipoprotein receptor in hepatic cells, is crucial for clearing cholesterol. Manuel et al. revealed that knock out LDLR of female mice are particularly prone to HFD-induced NAFLD (Garcia-Jaramillo et al. 2019). We observed that liraglutide upregulated the decreased the mRNA levels of LDLR in T2DM mice. Recent study showed that ACC plays an important role in the progression of NAFLD and inhibitory of ACC could be an attractive approach to treating NASH (Alkhouri et al. 2020; Sheng et al. 2019). Though, the mRNA and protein levels of ACC were decreased in T2DM mice liver in our study, the acivity of ACC is more crucial for its regulation of lipid metabolism. AMPK is a key modulator of ACC activation. AMPK signaling pathway plays an important role in ameliorating NAFLD and also regulates mitochondrial homeostasis and promotes autophagy, which is related to insulin resistance (Anggreini et al. 2023; Fang et al. 2022; Wang et al. 2022a). Metformin inhibits hepatic gluconeogenesis through AMPK-dependent regulation of orphan nuclear receptor small heterodimer partner(Kim et al. 2008). Activated AMPK which phosphorylates ACC, inhibits cholesterol and fatty acid synthesis. Mice with alanine knock-in mutations both ACC1 (at Ser79) and ACC2 (at Ser212) mice have elevated lipogenesis and lower fatty acid oxidation, which contribute to the progression of insulin resistance, glucose intolerance and NAFLD (Fullerton et al. 2013). Our results clearly showed that the phosphorylation of ACC by AMPK activation was increased with liraglutide. Mechanistic target of Rapamycin (mTOR) is another key downstream molecule of APMK. Liraglutide had no effects on the phosphorylation levels of mTORC1 targets S6K and S6, leading us to futher study the role of AMPK/ACC axis in the regulation of NAFLD.

Ferroptosis is a type of nonapoptotic cell death induced by lipid peroxidation and is suppressed by ferroptosis inhibitors, such as Fer-1, liproxstatin and DFO. We and others previously showed that ferroptosis is also an important factor in inducing NAFLD (Gao et al. 2021). Our current vivo study shows that Fer-1 almost fully reverse T2DM-induced liver damage without altering FBG. Fer-1 improved serum levels of ALT, TC and decreased liver fibrosis, hepatocytes size and binucleation of diabetic mice (Stancic et al. 2022). Some studies showed that ferroptosis may exacerbate liver fibrosis and liver injury, while ferroptosis might also inhibit the activation of hepatic stellate cells and thus ameliorate liver fibrosis (Feng et al. 2022). Further the role of ferroptosis in NAFLD should be better elucidate. In recent years, studies showed that some ferroptosis-related markers were founded, like PTGS2、4-HNE、MDA、TFR1 et al. (Li et al. 2020b; Stockwell 2022). 4-HNE, a lipid peroxidation marker offerroptosis, was significantly decreased in liver tissue of liraglutide treatment mice, but not cleaved caspase 3 which is marker of apoptosis. Thus, we speculate that liraglutide may be involved in the regulation of ferroptosis. Moreover, Fer-1 has shown stronger suppression of HG sensitivity on erastin or RSL3-induced cell death compared with other cell death inhibitors. Surpringly, liraglutide has a similar effect with Fer-1 on ferroptotic cell death in the presence of HG, indicating that liraglutide inhibits ferroptosis promoted by HG. Mechanistically, a recent study showed liraglutide treatment can change GPX4, SLC7A11 and TFRC protein levels during HG-induced HepG2 cell (Song et al. 2022a), whereas in our study liraglutide does not affect the expression of proteins involved in the ferroptosis regulation like TFRC, SLC7A11, ACSL4 and GPX4. We noticed that the HG concentration (75 mM) and liraglutide concentration (100 nM) used in this study was much lower than that used in our study (125 mM and 2.0 µM). It is possible that HG and liraglutide at such high concentrations may not be sensitive to these proteins. System Xc-glutathione (GSH)-GPX4 pathway is the main ferroptosis prevention system. On one hand, the sensitivity of ferroptosis changes with the alteration of GPX4. On the other hand, increase or decrease in GPX4 during ferroptosis depends on many regulations and factors. For example, study showed that GPX4 protein levels was significantly decreased when UMRC6 cells were treated with erastin and C2C12 cells were treated with RSL3 (Wang et al. 2021; Zhang et al. 2021). Furthermore, high glucose did not increase intracellular Fe^2+^ levels, and liraglutide didn’t significantly decrease intracellular Fe^2+^ levels, suggesting that at least in HepG2 cell line we have examined, high glucose sensitizing ferroptosis is at least not triggered by regulating iron concentration. However, our study showed that liraglutide upregulated the phosphorylation level of AMPK and ACC of HG-damaged cells, guiding us to investigate its role in the regualtion of ferroptosis by liraglutide. A recent study showed that glucose starvation largely rescued erastin-induced ferroptosis in immortalized mouse embryonic fibroblasts, and AMPK deletion promoted erastin-induced ferroptosis (Lee et al. 2020). In our study, we have used erastin or RSL3-induced ferroptotic cell death in the presence of HG, which could be rescued by liraglutide. Indeed, AMPK inhibition by compound C combined with ferroptosis inducers significantly decreased AMPK activation, while liraglutide partly reversed lower basal AMPK phosphorylation. All these results demonstrated that liraglutide partially inhibits ferroptosis through activating AMPK. It’s known that NAFLD is a heterogeneous metabolic disease. Studies showed that the improvement of NAFLD by liraglutide is associated with modulating gut microbiota and decreasing hepatic inflammation (Ipsen et al. 2018; Moreira et al. 2018). The association between gut microbiota, inflammation and ferroptosis should be further studied.

One unanwsered question in our study is whether liraglutide activates AMPK via hepatic GLP-1R. Several studies have demonstrated that GLP-1R isn’t expressed in hepatocytes(Panjwani et al. 2013). However, liraglutide is reported to bind to GLP-1 receptor in extrahepatic organs to stimulate hepatic FGF21 expression and activate the AMPK pathway (Liu et al. 2021; Salminen et al. 2017). Thus, the exact role of GLP-1R in liraglutide-mediated ferroptosis inhibition and NAFLD treatment remains further exploration, in which GLP-1R deficient mouse could be an powerful tool to solve this question. 3-hydroxy-3-methyl-glutaryl-coenzyme A reductase (HMGCR) is major rate-limiting enzyme in the mevalonate (MVA) pathway pathway, which is the most important metabolic pathway by which synthesize cholesterol (Huang et al. 2021). Inhibition the expression of HMGCR downregulate the mevalonate (MVA) pathway and glutathione peroxidase 4 (GPX4), thereby inducing cancer cell ferroptosis (Yao et al. 2021). Our study showed that the mRNA levels of HMGCR was decreased in T2DM-induced NAFLD mice, while liraglutide upregulated it. Whether liraglutide upregulated HMGCR is related to inhibiting ferroptosis should be further explored.

## Conclusions

In summary, our study reveals that AMPK/ACC inhibition dramatically promotes ferroptosis in T2DM-induced NAFLD, and suggests that inhititory ferroptosis is responsible for anti-NAFLD effects. These findings not only identify the mechanism of that liraglutide ameliorates T2DM-induced NAFLD by inhibiting ferroptosis via activation AMPK/ACC signaling, but also provide a new avenue for the treatment of T2DM-associated NAFLD.

### Supplementary Information

**Additional file 1: Table S1.** RNA-seq.

## Data Availability

Data and materials used and/or analyzed during the current study are available from the corresponding authors on reasonable request.

## Abbreviations

T2DM: Type 2 diabetes mellitus
NAFLD: Non-alcoholic fatty liver disease
AMPK: AMP-activated protein kinase
ACC: Acetyl-CoA carboxylase
4-HNE: 4-Hydroxynonenal
NASH: Nonalcoholic steatohepatitis
GLP-1: Glucagon-like peptide-1
GLP-1R: GLP-1 receptor
FBG: Fasting blood glucose
BW: Body weight
FGF21: Hepatic fibroblast growth factor 21
HFD: High-fat diet
IPTGG: Glucose tolerance test
ITT: Insulin tolerance test
AUC: Area under the curve
TC: Total cholesterol
TG: Triglyceride
LDL-C: Low density lipoprotein-cholesterol
ALT: Alanine aminotransferase
AST: Aspartate aminotransferase
H&E: Hematoxylin–eosin
SD: Standard deviation
HOMA-IR: Homeostasis model assessment of insulin resistance
GO: Gene Ontology
KEGG: Kyoto Encyclopedia of Genes and Genomes
IHC: Immunohistochemistry
LDLR: Low-density lipoprotein receptor
MTOR: Mechanistic target of Rapamycin
HMGCR: 3-Hydroxy-3-methyl-glutaryl-coenzyme A reductase
MVA: Mevalonate
GPX4: Glutathione peroxidase 4
